# VSP-2 attenuates secretion of inflammatory cytokines induced by LPS in BV2 cells by mediating the PPARγ*/*NF-κB signaling pathway

**DOI:** 10.1515/biol-2022-0861

**Published:** 2024-04-20

**Authors:** Jingxin Cui, Liwei Xu, Yimeng Sun, Lingfei Dai, Yuxiu Mo, Keli Yun, Yifei Chen, Linglin Chen

**Affiliations:** School of Pharmacy, Guilin Medical University, No. 1 Zhiyuan Road, Guilin, Guangxi, 541199, China; Scientific Experiment Center, Guilin Medical University, No. 1 Zhiyuan Road, Guilin, 541199, Guangxi, China

**Keywords:** neuroinflammation, VSP-2, proinflammatory cytokines, NF-κB pathway, PPARγ

## Abstract

Neuroinflammation, characterized by microglial activation and the subsequent secretion of inflammatory cytokines, plays a pivotal role in neurodegenerative diseases and brain injuries, often leading to neuronal damage and death. Alleviating neuroinflammation has thus emerged as a promising strategy to protect neurons and ameliorate neurodegenerative disorders. While peroxisome proliferator-activated receptor gamma (PPARγ) agonists have demonstrated potential therapeutic actions on neuroinflammation, their prolonged use, such as with rosiglitazone, can lead to cardiac risks and lipid differentiation disorders. In this study, we investigated the effects of a newly synthesized PPARγ agonist, VSP-2, on secretion of inflammatory cytokines in BV2 cells. Treatment with VSP-2 significantly reduced the mRNA and protein levels of proinflammatory cytokines such as interleukin-1β (IL-1β), IL-6, and tumor necrosis factor-α (TNF-α). Furthermore, VSP-2 attenuated the phosphorylation of nuclear factor kappa B (NF-κB) (65 kD) and IκBα, as well as the nuclear translocation of NF-κB (65 kD). Additionally, the use of PPARγ small interfering RNA was able to attenuate the effects of VSP-2 on proinflammatory cytokines and the NF-κB pathway. In conclusion, our findings suggest that VSP-2 effectively suppressed the expressions of IL-1β, IL-6, and TNF-α via the PPARγ/NF-κB signaling pathway. Given its potential therapeutic benefits, VSP-2 may emerge as a promising candidate for the treatment of neurodegenerative diseases or brain injuries associated with neuroinflammation.

## 1 Introduction

Neuroinflammation, a common occurrence in numerous neurodegenerative disorders, such as Parkinson’s disease and Alzheimer’s disease (AD) [1,2,3], is a primary contributor to neuronal damage. Microglia, the immune cells, and “defenders” in the brain, play a key role in mediating inflammation in the central nervous system (CNS) [4]. In neurodegenerative diseases, microglia become activated and release inflammatory cytokines, facilitating the clearance of harmful factors, such as amyloid plaques in AD [5,6]. This mechanism mitigates neuroinflammation during neurodegeneration. Nevertheless, the persistent presence of protein misfolding, aggregation, and activation throughout the progression of these diseases leads to sustained neuroinflammation [6,7,8]. Consequently, microglia release increased levels of proinflammatory cytokines, resulting in neuronal damage [8,9]. Therefore, the reduction in proinflammatory cytokines in neuroinflammation is considered a viable approach to mitigating brain damage and a strategy for the treatment of neurodegenerative diseases [10,11].

The production of proinflammatory cytokines and neuroinflammation is regulated by numerous signaling pathways [12,13]. Evidence is accumulating that the classical nuclear factor kappa B (NF-κB) pathway is chronically active in neuroinflammation, promoting the expression of proinflammatory cytokines [14,15]. When exposed to bacterial lipopolysaccharides (LPS) or other cellular stimuli, NF-κB complex, a dimer of p50 and p65, becomes activated and translocated to the cell nucleus, thereby inducing the production of proinflammatory cytokines [16,17]. Conversely, suppressing the NF-κB pathway can attenuate the production of proinflammatory cytokines and mitigate the progression of inflammation in CNS [18,19].

Rosiglitazone (Ros), an antidiabetic drug, has demonstrated the potential to ameliorate many neuroinflammatory diseases by downregulating the expression of proinflammatory factors [20,21]. This is achieved by activating the peroxisome proliferator-activated receptor gamma (PPARγ), inhibiting NF-κB binding to the promoters of proinflammatory genes [22]. However, the long-term use of Ros is associated with high risks of heart failure, myocardial infarction, and weight gain, limiting its application in inflammatory diseases [23,24,25]. These observed side effects are believed to be linked to the mechanism by which Ros activates PPARγ [26]. To address these limitations, a new PPARγ agonist, activating through an alternative mechanism, could be a superior therapeutic for neuroinflammation. SR1664, a PPARγ inhibitor, binds to a distal region of the PPARγ ligand binding pocket, distinct from the binding domain of Ros [26,27]. By leveraging the binding advantages of SR1664 and mitigating the binding disadvantages of Ros, we designed and synthesized VSP-2. The aim of this research is to explore the impact of VSP-2 on the suppression of inflammatory cytokine secretion in BV2 cells, along with its underlying mechanism, particularly involving PPARγ and the NF-κB pathway.

## 2 Methods

### 2.1 Reagents

VSP-2 (C_21_H_16_ClFN_4_O, MW: 394.83, purity ≥98%) was synthesized by our group, while Ros (purity ≥98%) and LPS were purchased from Sigma Aldrich (USA). Fetal bovine serum (FBS) was obtained from AusgeneX Pty Ltd (Australia), penicillin–streptomycin solution, thiazolyl blue tetrazolium bromide (MTT), dimethyl sulfoxide (DMSO), and Lipo8000™ Transfection Reagent were obtained from Beyotime Biotechnology (China). Other chemicals of analytical reagent grade were obtained from Shanghai Aladdin Biochemical Technology (China).

### 2.2 Cell culture

The mouse microglia BV2 cells were provided by the laboratory of Yuehan Zhou and purchased from BeNa Culture Collection (China). BV2 cells were cultured in DMEM medium (Thermo Fisher Scientific, USA) with a supplement of 10% FBS and 1% penicillin–streptomycin solution. The cells were maintained in a humidified incubator with an atmosphere of 5% CO_2_/95% air at 37°C.

### 2.3 Cell viability assay

BV2 cells (9,000 cells per well) were seeded into 96-well plates and allowed to adhere approximately for 15–18 h. Subsequently, cells were exposed to nine different concentrations of VSP-2 (0.01–100 μM) for 12 or 24 h. After incubation with MTT solution (0.5%) for 4 h, the supernatant was removed, and formazan crystals within the cells were completely dissolved with DMSO (150 μL). The absorbance was measured at wavelength of 490 nm using a microplate spectrophotometer.

### 2.4 Cell transfection

BV2 cells (9.5 × 10^5^ cells per well) were seeded in 6-well plates and allowed to reach 70–80% confluency overnight before transfection. For each well, a mixture containing 100 pmol specific small interfering RNA (siRNA) for PPARγ, 4 μL of Lipo8000 transfection reagent, and 125 μL of Opti-MEM Medium was added to the cells for 6 h.

The sequences of PPAR siRNA were as followed:

*PPAR*γ *1*, F: 5′-GCGGAGAUCUCCAGUGAUATT-3′

    R: 5′-UAUCACUGGAGAUCUCCGCTT-3′

*PPAR*γ *2*, F: 5′-GCAAGAGAUCACAGAGUAUTT-3′

    R: 5′-AUACUCUGUGAUCUCUUGCTT-3′

*PPAR*γ *3*, F: 5′-GGGCGAUCUUGACAGGAAATT-3′

    R: 5′-UUUCCUGUCAAGAUCGCCCTT-3′

### 2.5 Reverse transcription-qPCR

BV2 cells or transfected cells were treated with diverse concentrations (1, 3, and 10 μM) of VSP-2 or Ros (Ros, 1 μM) for 12 h after LPS (0.1 μg/mL) stimulation for 24 h. Total RNA was obtained using Trizol reagent (TIANGEN Biotech, China). Reverse transcription was performed using RTIII Super Mix with dsDNase kit (Mona Biotech, China). Quantitative real-time PCR was performed using MonAmp^™^ SYBR^®^ Green qPCR Mix (Mona Biotech, China) and a CFX96 Touch instrument (BioRad, USA). The gene expressions were calculated by 2^−ΔΔCt^ method [28] and normalized to the internal control *GAPDH*.

The primer sequences were as followed:

*GAPDH*, F: 5′-AGGTCGGTGTGAACGGATTTG-3′

    R: 5′-GGGGTCGTTGATGGCAACA-3′

*IL-1*β, F: 5′-AACTCAACTGTGAAATGCCACC-3′,

    R: 5′-CATCAGGACAGCCCAGGTC-3′

*IL-6*, F: 5′-TACTCGGCAAACCTAGTGCG-3′

    R: 5′-GTGTCCCAACATTCATATTGTCAGT-3′

*TNF-*α, F: 5′-CACCACGCTCTTCTGTCTACTG-3′

    R: 5′-GCTACAGGCTTGTCACTCGAA-3′

*PPAR*γ, F: 5′-CAAGCCCTTTACCACAGTTGA-3′

    R: 5′-CAGGTTCTACTTTGATCGCACTT-3′

### 2.6 Western blot analysis

BV2 cells or transfected cells were treated with VSP-2 or Ros for 12 h following LPS stimulation for 24 h. Protein extracts of BV2 cells were harvested by treating them with cold RIPA lysis buffer (Beijing Solarbio Science & Technology, China), supplemented with 1% PMSF (Beijing Solarbio Science & Technology, China) and phosphatase inhibitor (Sigma-Aldrich, USA). Cytosolic and nuclear protein fractions of BV2 cells were isolated using the Nuclear and Cytoplasmic Protein Extraction Kit (Beyotime Biotechnology, China). After being quantified with a BCA protein assay kit (Beyotime Biotechnology, China), proteins (20 μg per lane) were resolved on either an 8% SDS-PAGE gel for proteins below 30 kD or a 10% SDS-PAGE gel for larger proteins. Following transfer and blocking with 5% skim milk, the PVDF membrane (Millipore, USA) was incubated overnight at 4°C with primary antibodies against PPARγ, interleukin-1β (IL-1β), interleukin-6 (IL-6), tumor necrosis factor-α (TNF-α) (1:500, Beyotime Biotechnology, China), GAPDH (1:10,000, Abcam, USA), phospho-NF-κB (65 kD), phospho-IκBα, NF-κB (65 kD), IκBα, PCNA (1:1,000, Cell Signaling Technology, USA). Subsequently, the membrane was incubated with relative HRP-labeled secondary antibody (1:1,000, Beyotime Biotechnology, China) for 2 h. Bands were visualized using immobilon enhanced chemiluminescence ultra western HRP substrate (Millipore, USA) and quantified with ImageJ software (v1.8.0, NIH, USA).

### 2.7 Immunofluorescence

BV2 cells (1.2 × 10^4^ cells per well) were seeded onto coverslips placed in a 24-well plate and allowed to adhere for 15–18 h. Subsequently, the cells were treated with LPS, VSP-2, or Ros and fixed with 4% paraformaldehyde (PFA) for 20 min. Prior to immunostaining, the cells were permeabilized with 0.3% Triton X-100/PBS solution for 20 min and blocked with 3% FBS for 1.5 h. The cells were then incubated with an antibody against NF-κB (p65, 1:100) for 15–18 h at 4°C and Alexa Fluor 594-labeled second antibody (1:200, Beyotime Biotechnology, China) for 1.5 h at 37°C. After counterstaining with DAPI (Beyotime Biotechnology, China), the immunofluorescence signals were immediately visualized using a fluorescence microscopy (IX73, Olympus, Japan).

### 2.8 Statistical analysis

All data were expressed as the mean value ± standard deviation (SD). Statistical comparisons between two groups were performed using *t*-test, and comparisons among multiple groups were performed using one-way ANOVA analysis, followed by Tukey’s test with GraphPad Prism software (v6.0 for Windows, GraphPad Software, USA). *P* < 0.05 was considered a significant statistical difference.

## 3 Results

### 3.1 Steps for synthesis of VSP-2

**Figure 1:**
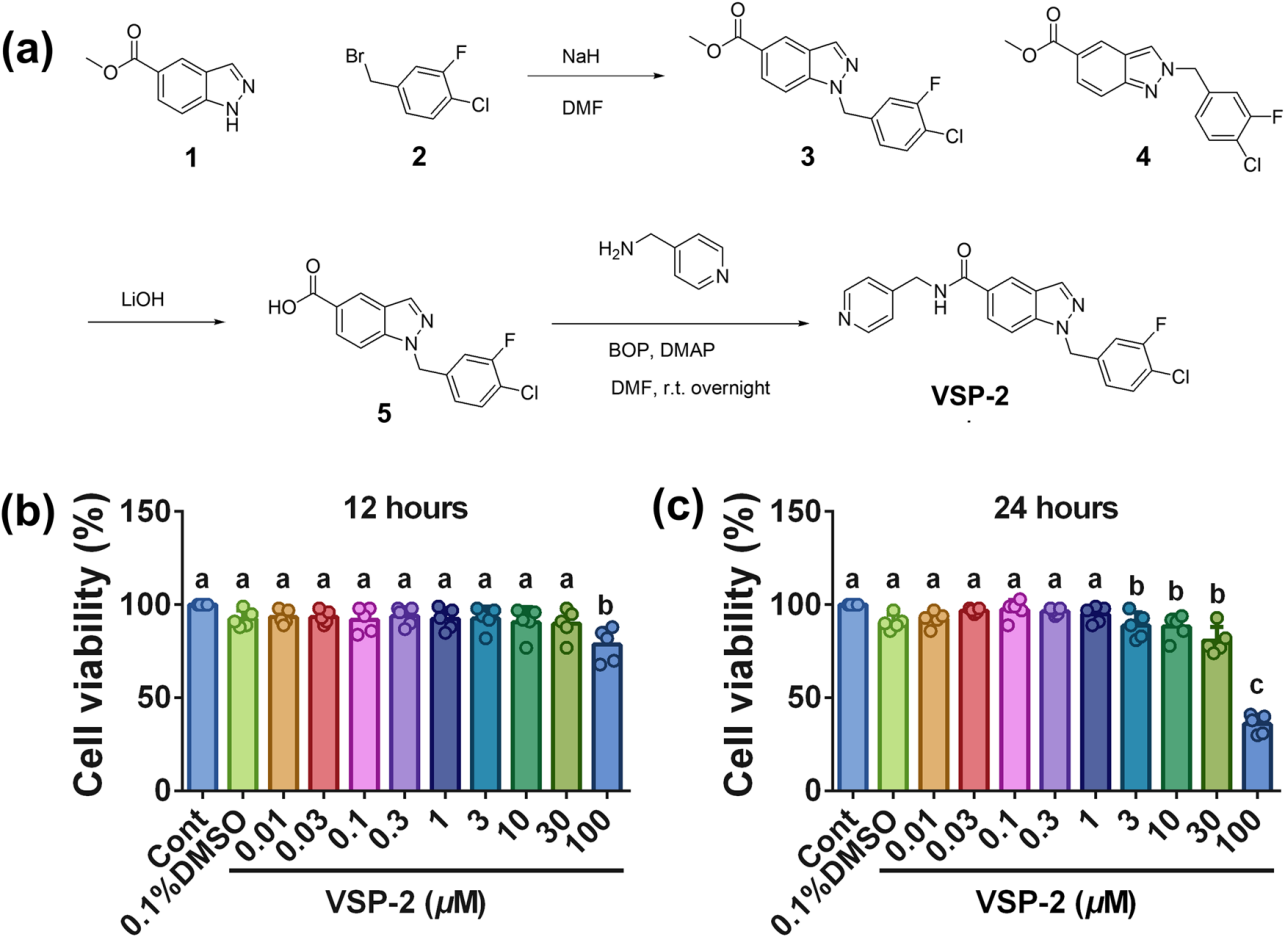
The synthesis steps of VSP-2 and the effect of different concentrations of VSP-2 on the viability of BV2 cells. (a) The synthesis of VSP-2. (b) The effect of VSP-2 on BV2 cells viability after 12 h. BV2 cells were treated with VSP-2 (0.01, 0.03, 0.1, 0.3, 1, 3, 10, 30, and 100 μM). (c) The effect of VSP-2 on BV2 cells viability after 24 h. Identical letters indicate non-significant differences (*P* > 0.05), while different letters indicate significant differences (*P* < 0.05), *n* = 5.

The synthesis of VSP-2 was achieved through three steps (Figure 1a). In Step I, sodium hydride (NaH, 850 mg, 60%) was added to a solution of methyl 1*H*-indazole-5-carboxylate (compound 1, 12.5 g) dissolved in *N,N*-dimethylformamide (DMF, 60 mL) at 0°C. Following a 30-min stirring period under an N_2_ atmosphere, a solution of 4-(bromomethyl)-1-chloro-2-fluorobenzene (compound 2, 4.7 g) in DMF (20 mL) was added, and the mixture was mixed overnight at 20–25°C. Subsequently, the reaction was quenched with saturated NH_4_Cl solution and extracted with ethyl acetate (120 mL). The layer of ethyl acetate was separated, dried over anhydrous sodium sulfate (Na_2_SO_4_), filtered, and evaporated. Two compounds, methyl 1-(4-chloro-3-fluorobenzyl)-1*H*-indazole-5-carboxylate (compound 3, 1.5 g) and methyl 2-(4-chloro-3-fluorobenzyl)-2*H*-indazole-5-carboxylate (compound 4, 560 mg) were obtained after the remainder was purified and eluted with petroleum ether/ethyl acetate (10:1 to 4:1) by a silica gel column. In Step II, compound 3 (1.5 g) was added to LiOH (790 mg, 18.9 mmol) in tetrahydrofuran/H_2_O/methanol (1:1:1, 30 mL). The reaction mixture was stirred overnight at a temperature of 20–25°C. Subsequently, the mixture was acidified to pH = 5 using a 1 mol/L HCl solution and extracted with ethyl acetate (100 mL). The compound 1-(4-chloro-3-fluorobenzyl)-1*H*-indazole-5-carboxylic acid (compound 5, 1.2 g, 85.0% yield) was obtained after being separated, dried, and evaporated. In Step III [29,30], pyridin-2-ylmethanamine (620 mg) was added to a mixture containing compound 5 (1.2 g) dissolved in DMF (30 mL). Subsequently, Castro’s reagent (BOP, 3.5 g) and 4-dimethylaminopyridine (DMAP, 100 mg) were added. The reaction mixture was stirred overnight at 20–25°C. After this, the mixture was extracted with ethyl acetate (80 mL) and washed twice with saline (50 mL each). The crude product was purified by silica gel column chromatography using a dichloromethane/methanol gradient (50:1 to 20:1). After separation, drying, and evaporation, the compound 1-(4-chloro-3-fluorobenzyl)-*N*-(pyridin-4-ylmethyl)-1*H*-indazole-5-carboxamide (650 mg, yield 41.2%, VSP-2) was obtained as a white solid.

^1^H NMR (400 MHz, DMSO) *δ* 9.17 (t, *J* = 5.9 Hz, 1H), 8.51 (d, *J* = 5.9 Hz, 2H), 8.42 (s, 1H), 8.32 (s, 1H), 7.95 (dd, *J* = 8.9, 1.5 Hz, 1H), 7.84 (d, *J* = 8.9 Hz, 1H), 7.54 (t, *J* = 8.1 Hz, 1H), 7.31 (dd, *J* = 14.7, 3.9 Hz, 3H), 7.04 (dd, *J* = 8.3, 1.3 Hz, 1H), 5.74 (s, 2H), 4.53 (d, *J* = 5.9 Hz, 2H).

### 3.2 Effect of VSP-2 on BV2 cells’ viability

The MTT assay was performed to investigate the impact of VSP-2 on the viability of BV2 cells. Cells were exposed to varying concentrations of VSP-2 (ranging from 0.01 to 100 μM) or DMSO (0.1%) for durations of 12 or 24 h. The results indicated a reduction in BV2 cells viability upon treatment with 100 μM VSP-2 for 12 h (*P* < 0.001, Figure 1b) and concentrations ranging from 3 to 100 μM for 24 h (Figure 1c). To mitigate the effects of cell death induced by higher VSP-2 concentrations or prolonged exposure, concentrations of 1, 3, and 10 μM for 12 h were selected for subsequent experimentation, limited to a duration of 12 h.

### 3.3 VSP-2 exerted a concentration-dependent effect on the production of proinflammatory cytokines

**Figure 2:**
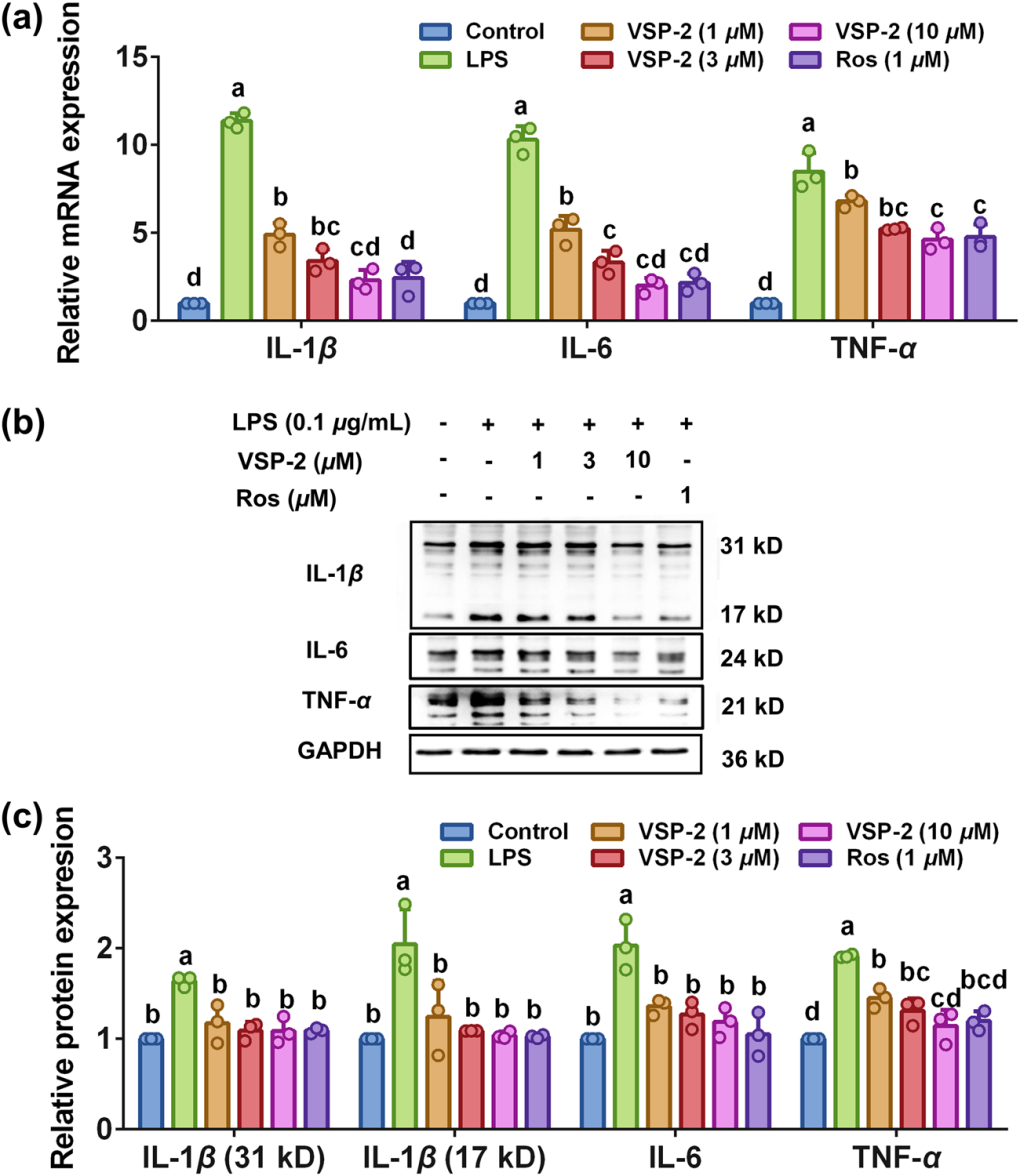
VSP-2 downregulated the levels of IL-1β, IL-6, and TNF-α mRNA and protein. BV2 cells were treated with VSP-2 (1, 3, and 10 μM) or Ros (1 μM) for 12 h after LPS stimulated for 24 h. Total mRNA and protein were obtained for RT-qPCR and Western blotting. (a) Up-regulated expressions of proinflammatory cytokines were diminished by VSP-2 (1, 3, and 10 μM) or Ros (1 μM). (b and c) Levels of proinflammatory cytokines were decreased by VSP-2 (1, 3, and 10 μM) or Ros (1 μM). Identical letters indicate non-significant differences (*P* > 0.05), while different letters indicate significant differences (*P* < 0.05), *n* = 3.

Elevated proinflammatory cytokines are hallmarks of neuroinflammation [31]. To explore the function of VSP-2 on LPS-induced BV2 cells, we quantified the expression levels of IL-1β, IL-6, and TNF-α using RT-qPCR and Western blot analysis. BV2 cells were pretreated with VSP-2 (1, 3, and 10 μM) or Ros (1 μM) for 12 h, followed by LPS stimulation (0.1 μg/mL) for 24 h. The significantly increased expression of IL-1β, IL-6, and *TNF-*α mRNA were repressed with different concentrations of VSP-2 or Ros. Notably, the effect of 10 μM VSP-2 on mRNA expression of these proinflammatory cytokines was comparable to that of 1 μM Ros. Furthermore, treatment with 10 μM VSP-2 or 1 μM Ros resulted in IL-1β and IL-6 mRNA levels nearing those of the control group (Figure 2a). Comparable to mRNA expression, the elevated protein levels of IL-1β (17 kD and 31 kD), IL-6, and TNF-α were also reduced by VSP-2 or Ros treatment (Figure 2b and c). The effect of VSP-2 at concentrations ranging from 3 to 10 μM on the protein expression of these proinflammatory cytokines was similar to that of 1 μM Ros. The data indicated a concentration-dependent inhibitory effect of VSP-2 on the upregulation of proinflammatory cytokines at both mRNA and protein levels. At a concentration of 10 μM, VSP-2 exhibited comparable effects to Ros.

### 3.4 VSP-2 blocked LPS-induced activation of NF-κB pathway

**Figure 3:**
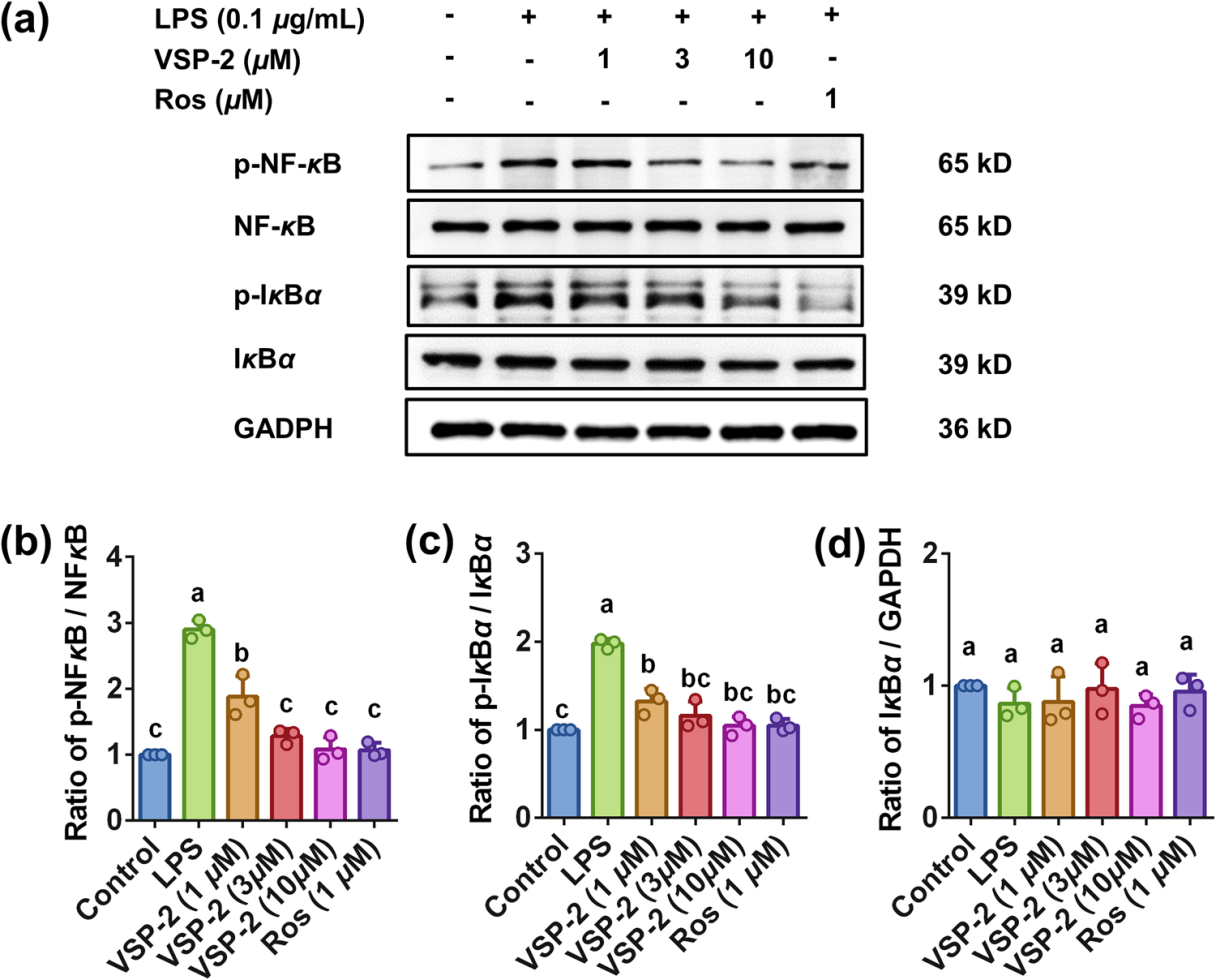
VSP-2 decreased the phosphorylation of NF-κB (65 kD) and IκBα proteins. Protein extracts were prepared for Western blot analysis after BV2 cells were stimulated with LPS for 24 h, followed by treatment with varying concentrations (1, 3, and 10 μM) of VSP-2 or Ros (1 μM) for 12 h (a) Blots of p-NF-κB (65 kD), NF-κB (65 kD), p-IκBα, IκBα, and GAPDH in BV2 cells. (b) VSP-2 reduced the phosphorylation of NF-κB. (c) VSP-2 reduced the phosphorylation of IκBα. (d) Levels of IκBα protein were unchanged in each group. Identical letters indicate non-significant differences (*P* > 0.05), while different letters indicate significant differences (*P* < 0.05), *n* = 3.

Evidence indicates that NF-κB pathway has been activated during neuroinflammation [32]. To assess whether treatment with VSP-2 can attenuate the activation of the NF-κB pathway, we examined the protein expression of phosphorylated NF-κB (65 kD), total NF-κB (65 kD), phosphorylated IκBα, and total IκBα in BV2 cells using Western Blot analysis. Our findings reveal that both increasing concentrations of VSP-2 and a constant concentration of 1 μM Ros suppressed phosphorylation of IκBα and NF-κB. At concentrations of 3 and 10 μM, these treatments effectively abrogated LPS-induced phosphorylation, returning levels to those observed in the control group (Figure 3a–c). In contrast, the protein level of IκBα remained relatively unchanged across all groups following LPS stimulation (Figure 3d).

**Figure 4:**
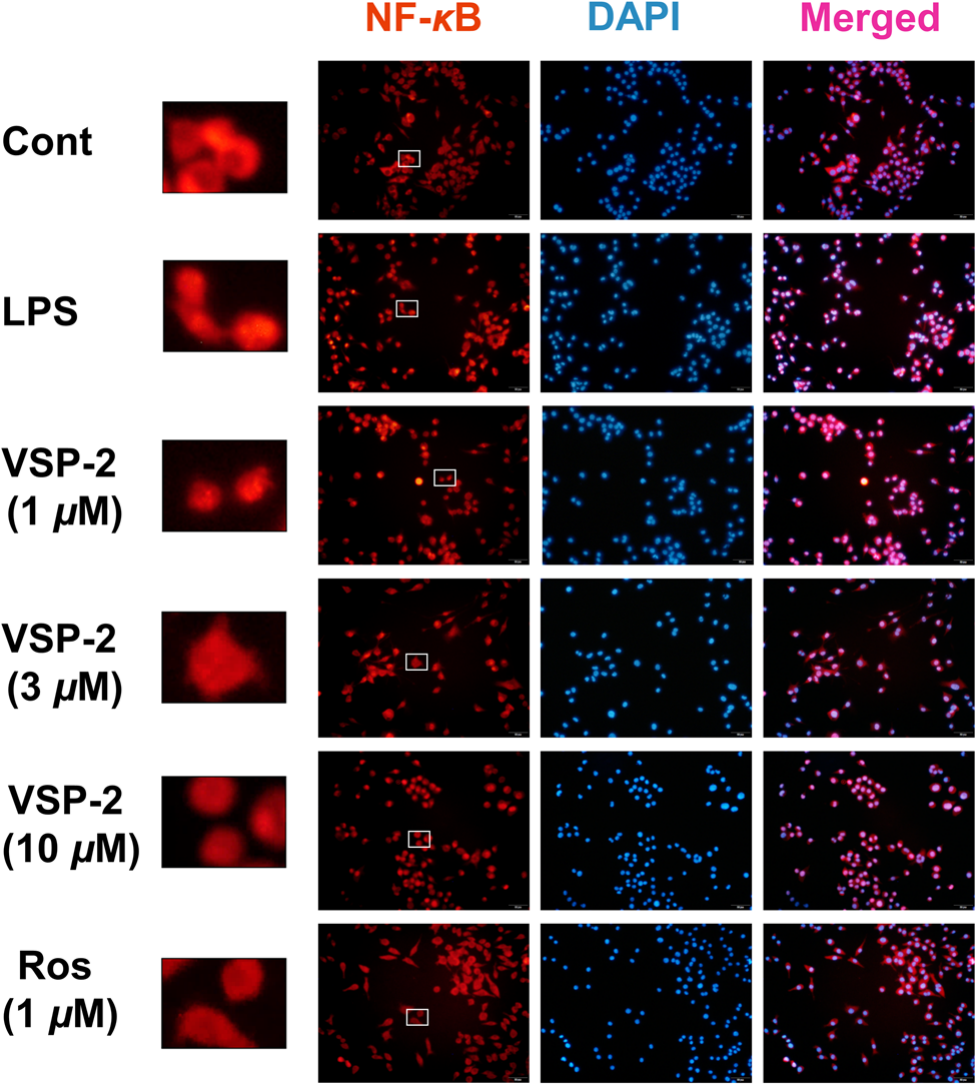
NF-κB nuclear translocation was inhibited by VSP-2 (40×). BV2 cells were treated with different concentrations (1, 3, and 10 μM) of VSP-2 or Ros (1 μM) for 12 h after LPS stimulation for 24 h. Cells were stained for immunofluorescence staining. Red: NF-κB (p65), blue: DAPI. Scale bar 20 μm.

Once activated outside the nucleus, NF-κB complex translocate into the nucleus to regulate gene expression [33,34]. To assess the impact of VSP-2 on NF-κB pathway, we employed immunofluorescence to detect the NF-κB nuclear translocation. Our findings indicate that NF-κB nuclear translocation induced by LPS stimulation was progressively suppressed by increasing concentrations of VSP-2 (Figure 4). Notably, at a concentration of 10 μM, VSP-2 exerted a similar inhibitory effect to 1 μM Ros. These results suggested that VSP-2 blocked the activation of the NF-κB pathway triggered by LPS. Furthermore, at a concentration of 10 μM, the inhibitory effect of VSP-2 is comparable to that of 1 μM Ros.

### 3.5 PPARγ siRNA reversed the function of VSP-2 on the expression of proinflammatory cytokines

**Figure 5:**
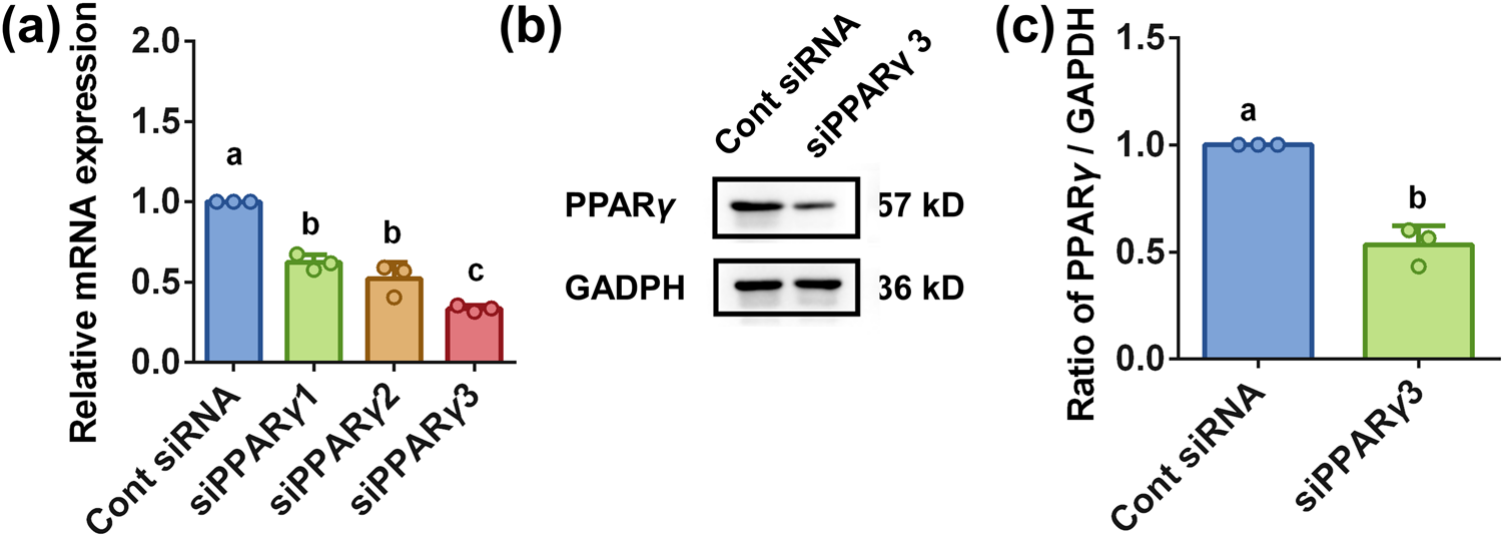
siPPARγ 3 downregulated PPARγ mRNA and protein expression. Control siRNA or siPPARγ was applied to BV2 cells for 6 h. Total mRNA or protein was obtained 36 or 48 h after transfection, respectively. (a) siPPARγ 3 exhibited greater silencing efficiency than siPPARγ 1 and 2. (b and c) PPARγ protein level was decreased after transfection with siPPARγ 3. Identical letters indicate non-significant differences (*P* > 0.05), while different letters indicate significant differences (*P* < 0.05), *n* = 3.

To confirm the function of PPARγ in downregulation of proinflammatory cytokines mRNA and protein by VSP-2, we transfected BV2 cells with control siRNA or PPARγ siRNA (siPPARγ) for 6 h. Total mRNA or protein was harvested for RT-qPCR or Western blot analysis 36 or 48 h after transfection respectively. As shown in Figure 5a, siPPARγ 3 exhibited the highest silencing efficiency. Consistent with this, PPARγ protein levels were significantly reduced following transfection with siPPARγ 3 (Figure 5b and c). Therefore, siPPARγ 3 was chosen for subsequent experiments.

**Figure 6:**
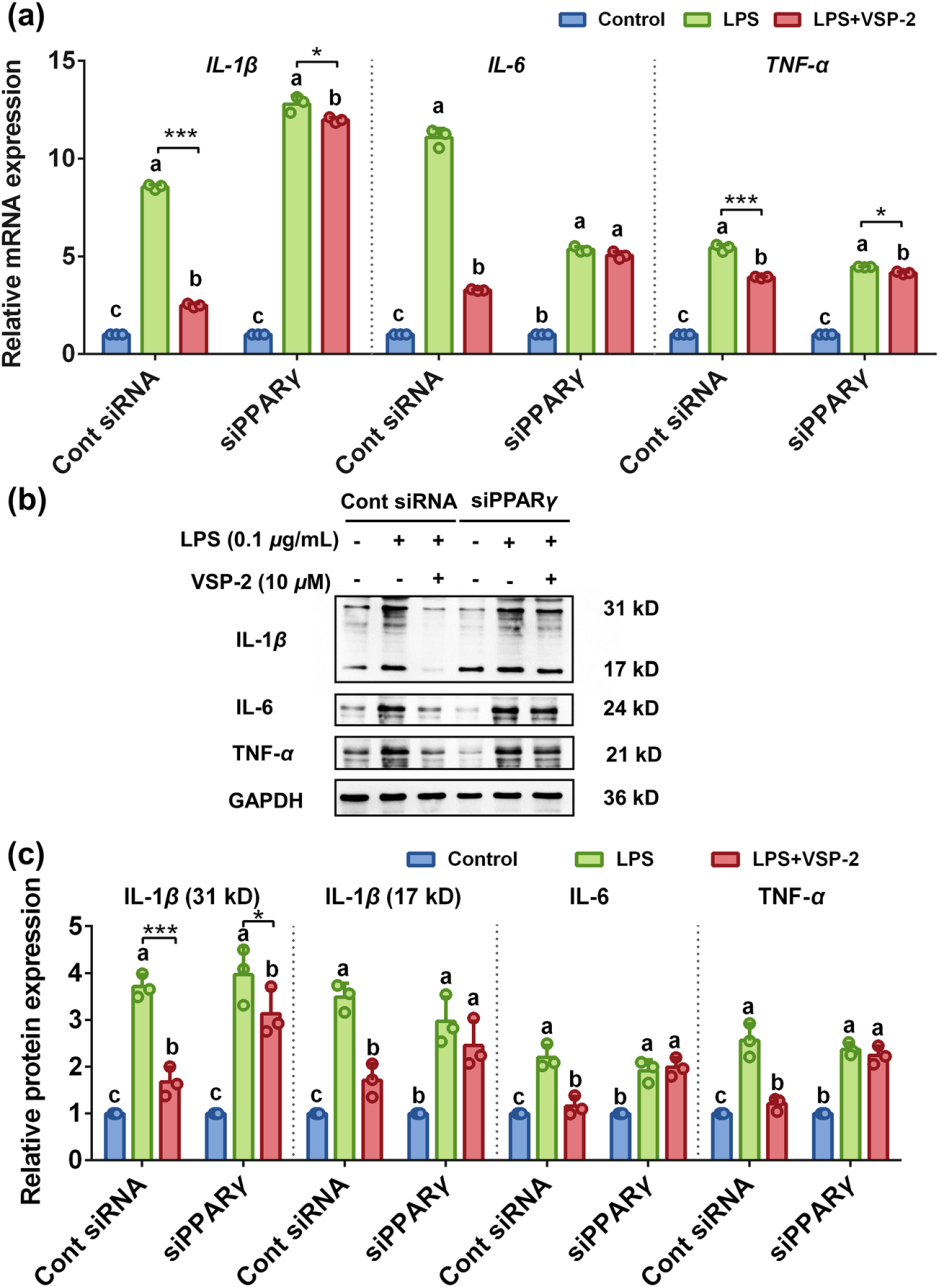
Knockdown of PPARγ reversed the suppressive effect of VSP-2 on the expression of proinflammatory cytokines. BV2 cells were transfected with siPPARγ for 6 h, then stimulated with LPS for 24 h, followed by treatment with 10 μM VSP-2 for an additional 12 h. (a) Knockdown of PPARγ abolished the downregulation of proinflammatory cytokines mRNA by VSP-2. (b and c) Knockdown of PPARγ reversed the inhibitory effect of VSP-2 on the protein levels of proinflammatory factors. Identical letters indicate non-significant differences (*P* > 0.05), while different letters indicate significant differences (*P* < 0.05), ^*^ *P* < 0.05, ^***^ *P* < 0.001, *n* = 3.

After transfection with siPPARγ 3 (hereinafter referred to as siPPARγ), BV2 cells were stimulated with 0.1 μg/mL LPS and subsequently treated with 10 μM VSP-2. The mRNA levels of *IL-1*β, IL-6, and *TNF-*α were downregulated by VSP-2 treatment. However, this suppressive effect of VSP-2 on the mRNA expression of the proinflammatory cytokines was reversed by the knockdown of PPARγ using siPPARγ (Figure 6a). Furthermore, the protein levels of these cytokines, which were reduced by VSP-2, were also reversed by siPPARγ treatment (Figure 6b and c). These findings suggested that suppressive action of VSP-2 on the expression of proinflammatory cytokines was mediated through PPARγ.

### 3.6 PPARγ siRNA reversed the effect of VSP-2 on NF-κB pathway

**Figure 7:**
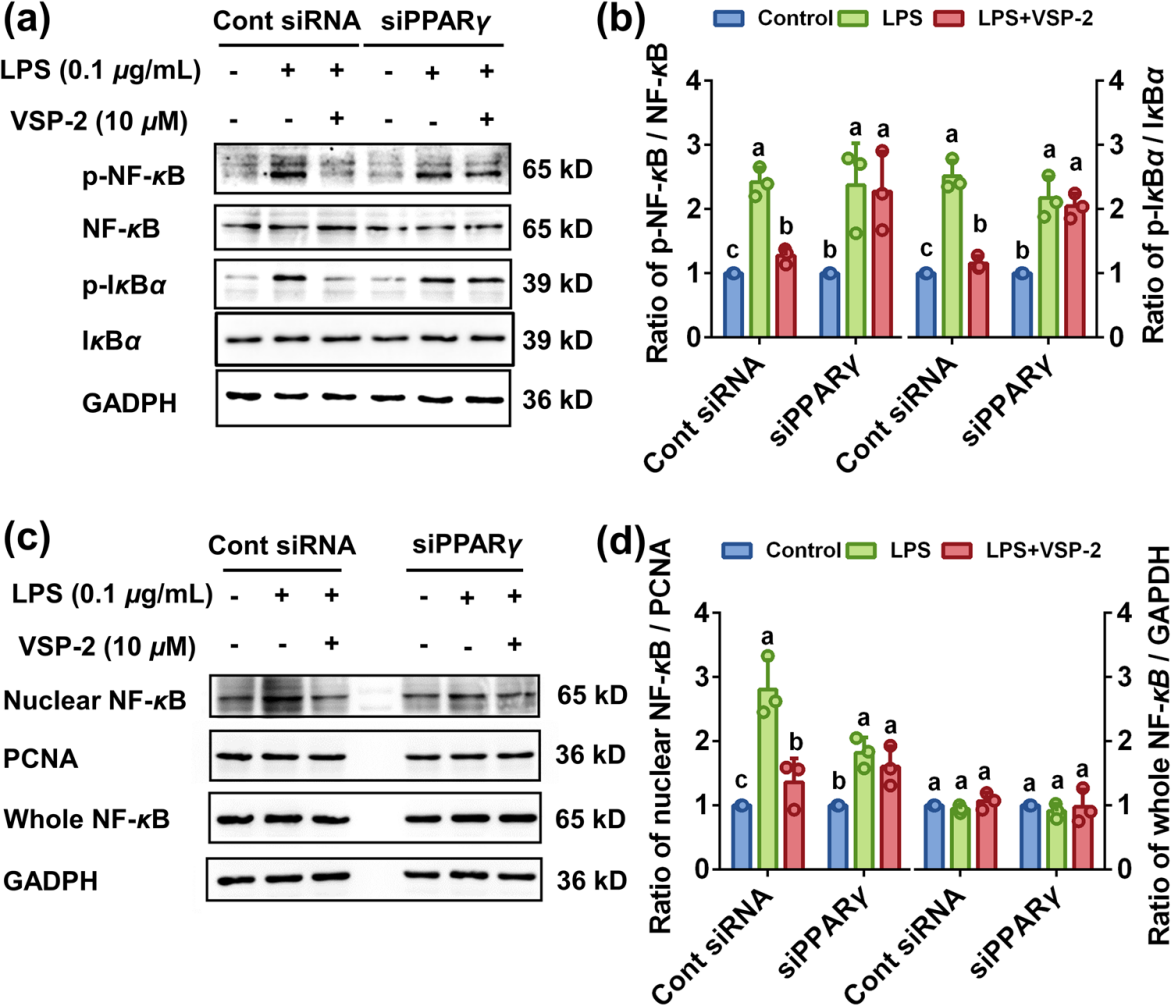
Knockdown of PPARγ reversed the inhibitory effect VSP-2 on the NF-κB signaling pathway. BV2 cells were transfected with siPPARγ for 6 h, followed by LPS stimulation for 24 h and subsequent treatment with 10 μM VSP-2 for 12 h. (a and b) Knockdown of PPARγ attenuated the inhibitory effect of VSP-2 on phosphorylation of NF-κB and IκBα. (c and d) Knockdown of PPARγ led to an increase in nuclear NF-κB. Identical letters indicate non-significant differences (*P* > 0.05), while different letters indicate significant differences (*P* < 0.05), *n* = 3.

To elucidate the mechanism by which PPARγ mediates the blockade of NF-κB signaling pathway by VSP-2, we quantified the protein levels of p-IκBα, p-NF-κB (65 kD), IκBα, NF-κB (65 kD), and nuclear NF-κB (65 kD) by Western blot analysis. Treatment with VSP-2 led to a decrease in the phosphorylation of both NF-κB (65 kD) and IκBα. However, the inhibitory effect of VSP-2 on phosphorylation of these proteins was reversed by knockdown of PPARγ (Figure 7a and b). Concurrently, an increase in nuclear NF-κB was observed upon transfection with siPPARγ, suggesting that the regulatory action of VSP-2 on nuclear NF-κB was reversed by PPARγ knockdown (Figure 7c and d). Collectively, these findings suggested that VSP-2 blocked the NF-κB pathway through PPARγ.

## 4 Discussion

Neuroinflammation encompasses the inflammatory response of the CNS, which is triggered by internal or external stimuli [2,35]. Persistent neuroinflammation can lead to neuronal damage and even affect the entire brain. Therefore, alleviating neuroinflammation may help to protect neurons and potentially impact the entire brain, emphasizing the need for therapeutic strategies to mitigate this inflammatory response. In the present study, we successfully synthesized VSP-2 and demonstrated its ability to alleviate production of neuroinflammatory cytokines in BV2 cells by downregulating the expression of proinflammatory cytokines. Furthermore, our findings suggest that VSP-2 decreases production of neuroinflammatory cytokines of BV2 cells by suppressing NF-κB pathway and activating PPARγ.

Drawing upon the structural advantages of SR1664 and the structural disadvantages of Ros, we designed VSP-2, which was synthesized through a series of reactions including amide bond formation, ester hydrolysis, and carbon-nitrogen coupling. Ros is known as a full agonist for PPARγ and typically binds to helix 12 (H12) of the PPARγ ligand binding domain, leading to the formation of an AF-2 motif that regulates gene transcription [26,36,37]. In contrast, SR1664 acts as a PPARγ inhibitor binding to PPARγ without directly interacting with H12 [26]. Given the structural similarities between VSP-2 and SR1664, we hypothesize that VSP-2 binds to PPARγ in a manner that is comparable to SR1664. However, this hypothesis remains to be further validated through subsequent studies.

Despite potential differences in the mechanisms of PPARγ activation between Ros and VSP-2, VSP-2 demonstrates a comparable ability to attenuate the increase in inflammatory cytokines following microglial activation. In our study, BV2 cells were stimulated with LPS to induce similar microglial response in neuroinflammatory reaction, leading to elevated expressions of IL-1β, IL-6, and TNF-α. Administration of VSP-2 or Ros resulted in reduced mRNA and protein levels of the proinflammatory factors. These findings suggest that, in the inflammatory response of neurodegenerative diseases, VSP-2 may also possess the ability to reduce inflammation, similar to Ros.

The mechanism underlying VSP-2’s ability to suppress inflammatory cytokines is analogous to Ros, involving the NF-κB signaling pathway. Evidence indicates that the hyperactivation of the NF-κB pathway plays a pivotal role in the inflammatory response, facilitating the release of proinflammatory cytokines [38,39]. In the normal state, the NF-κB complex, composed of a dimer of p50 (NF-κB1) and p65 (NF-κB 3 or RelA), remains bound to the inhibitory proteins of κB (IκBs) [40]. Upon exposure to bacterial LPS or other noxious cellular stimuli, IκB kinase (IKK) is activated, resulting in phosphorylation of the IκB proteins, such as IκBα [41]. Consequently, phosphorylated IκBs are isolated from the NF-κB complex and degraded by the proteasome [16]. This degradation process triggers the activation of the NF-κB complex, leading to its translocation to the nucleus and subsequent binding to the specific NF-κB DNA-binding sites, thereby promoting the production of proinflammatory cytokines [15,17]. Consistent with previous studies, we observed elevated levels of p-IκBα and p-NF-κB (65 kD) following LPS stimulation, indicating activation of the NF-κB pathway. Notably, treatment with VSP-2 or Ros resulted in decreased protein levels and NF-κB nuclear translocation, suggesting that VSP-2 may exert inhibitory effects on the NF-κB pathway comparable to Ros. Interestingly, we did not observe the degradation of IκBα following LPS exposure. This observation could be attributed to NF-κB feedback mechanisms leading to upregulated IκBα expression [42], or alternatively, VSP-2 may have a greater impact on NF-κB nuclear translocation.

The inhibitory effects of VSP-2 on NF-κB signaling pathway may be linked to PPARγ, a nuclear receptor involved in fat differentiation, glucose metabolism, and inflammatory responses [22,43]. PPARγ and its agonists have been reported to modulate neuroinflammation and are used as a nonspecific class of drugs in AD mouse models [44]. These agonists can stimulate PPARγ, promoting its binding to the p65 subunit in the nucleus, thereby inhibiting the NF-κB complex binding to the promoters of proinflammatory genes [45,46]. In our previous work, we developed VSP-2 as a PPARγ agonist, distinct from Ros in its mode of activation. Notably, both VSP-2 and Ros share similar abilities in reducing inflammatory cytokines and suppressing the NF-κB signaling pathway. To further investigate the role of PPARγ in VSP-2’s mechanism, we knocked down the PPARγ using a siPPARγ. Our results suggest that the effects of VSP-2 on proinflammatory factor expression and NF-κB signaling pathway were abolished after PPARγ knockdown. This finding verifies that the effect of VSP-2 on reducing proinflammatory factors and inhibiting NF-κB signaling pathway is PPARγ-dependent.

In the present study, we discovered that VSP-2 effectively reduces the release of inflammatory factors by activating PPARγ and inhibiting the NF-κB pathway, a mechanism similar to that of Ros. This suggests that VSP-2 has the potential to emerge as a viable alternative to Ros in the treatment of neuroinflammation associated with neurodegenerative disorders. However, our study has limitations. First, our focus has been primarily on the anti-inflammatory effects of VSP-2, yet confirmation of its agonistic activity on PPARγ is still outstanding. This could be addressed by performing molecular docking method or TR-FRET assay, as well as examining mRNA expression of CD36, a target gene of PPARγ. Second, although we have demonstrated VSP-2’s impact on NF-κB signaling pathway, further research is needed to ascertain whether it indeed triggers the binding of PPARγ to the p65 subunit in the nucleus. Finally, although VSP-2 attenuates inflammatory factors, further studies are required to assess its neuroprotective potential against neuronal damage.
